# Role of Yoga as Adjunctive Therapy for Migraines: A Narrative Review of the Literature

**DOI:** 10.7759/cureus.48434

**Published:** 2023-11-07

**Authors:** Meet Popatbhai Kachhadia, Zorain M Khalil, Sanjay Shah, Moiz Fawad, Hamza Sajjad, Kameshwar P Yadav, Neha R Kanthala, Tirath Patel, Uzoamaka C Egbujo, Basant K

**Affiliations:** 1 Internal Medicine, Pandit Deendayal Upadhyay (PDU) Medical College, Civil Hospital Campus, Rajkot, IND; 2 Medicine and Surgery, Combined Military Hospital, Lahore, PAK; 3 Medicine and Surgery, Janaki Medical College, Janakpur, NPL; 4 Neurological Surgery, King Saud Hospital, Unaizah, SAU; 5 Medicine, Aziz Bhatti Shaheed Teaching Hospital, Gujrat, PAK; 6 Internal Medicine, Universal College of Medical Sciences (UCMS), Bhairahawa, NPL; 7 Internal Medicine, Kamineni Academy of Medical Science and Research Centre, Hyderabad, IND; 8 Medicine, American University of Antigua, Saint John's, ATG; 9 Pediatrics, Lagos University Teaching Hospital, Lagos, NGA; 10 Medicine, Tanta University, Tanta, EGY

**Keywords:** integrative wellness, headache, complementary and integrative health, yoga, migraine

## Abstract

Migraines are chronic, painful, and one of the most prevalent disabling primary headache disorders, mainly treated with pharmacological methods. Patients suffering from migraine suffer from a significantly reduced quality of life. The use of non-pharmacological methods to reduce the stress and anxiety associated with long-term chronic conditions can help improve quality of life, reduce disease burden, and subsequently alleviate the economic burden on the patient. This review aims to discuss the use of yoga in patients with migraine headaches as a non-pharmacological method. We discuss the most recently published literature discussing the use of yoga as an add-on therapy for patients with migraines in order to reduce the severity of their symptoms, anxiety, and stress. Despite the presence of limitations and the need for further studies, the current data suggest that yoga can be beneficial in helping patients suffering from migraine headaches by reducing their frequency, duration, and pain. Yoga has also demonstrated improvement in the headache impact severity migraine disability assessment test.

## Introduction and background

Migraine remains one of the most widespread and incapacitating chronic neurological conditions. It is one of more than 200 types of headaches and, to date, remains the most common and most disabling [1]. Migraines are a primary headache disorder; they often occur in patients aged between 35 and 45 years, occurring more commonly in women. Migraines are recurrent and lifelong as well in some cases. Recent onset episodes are the most characteristic feature. They are caused when pain-mediating inflammatory substances are released into the brain [2]. These inflammatory substances surround the nerves and blood vessels of the brain, resulting in vasodilation and pain. Attacks are most commonly of moderate or severe intensity, one-sided, pulsating in quality, and aggravated by routine physical activity and have a duration of hours to two to three days [2]. Most individuals with migraine experience what is referred to as "episodic migraine," characterized by having <15 headache days in a month; research has indicated that approximately two-thirds of patients suffering from migraine have around four headache days in a month [3].

If a patient's illness is unusual, complex, or suddenly worsens, then diagnostic modalities such as a magnetic resonance imaging scan or a computerized tomography scan may be used to rule out other causes of pain. The management of migraine aims to curtail the symptoms and prevent further attacks. Several medications can be used, which are divided into two main categories: pain-relieving and preventive. Pain-relieving medications and acute treatments are used to stop symptoms. They include triptans, dihydroergotamine, lasmiditan, calcitonin gene-related peptide antagonists, zavegepant (nasal), opioids, and anti-nausea medications. Preventive medications are used to reduce the frequency or severity of migraine attacks. These include botox, atogepant, rimegepant, and certain classes of antidepressants; anti-seizure drugs; and blood pressure-lowering medications [4]. Medication-based prevention is typically recommended for patients encountering four or more headaches a month. Several lifestyle and home remedies are also recommended to help reduce symptoms such as relaxation techniques, a stable sleeping and eating routine, staying hydrated, and regular aerobic exercise. Adopting changes in lifestyle behavior presents an economical and easily accessible preventive approach suitable for individuals with a varying frequency of migraine attacks [5]. Current evidence suggests that disruptions in everyday routine result in worsening migraine attacks and that a consistent lifestyle is an effective behavioral preventive strategy for migraines. Creating effective recommendations for altering lifestyle behaviors should be grounded in evidence linking certain factors to migraine symptoms or associated disability [6].

Another important aspect is anxiety; migraines have been linked to stress and accompany stressful situations [7]. Improving the overall quality of life of patients with migraine headaches has been linked to effective management of stress and pain. Yoga is a practice that has undergone extensive scrutiny by the scientific community and gained acknowledgment for its potential to diminish stress [8,9]. Yoga has proved to reduce anxiety, stress, and depression, therefore resulting in an improved quality of life [10-12]. This also indicated its potential use in pain management in chronic illnesses, such as migraine headaches. Research demonstrates that methods used in yoga can contribute to the management of pain by reestablishing autonomic balance and mitigating the disability associated with chronic health conditions [13,14]. Therefore, utilizing a yoga-based approach can help alleviate the strain on healthcare resources that are currently stretched to their limits. Yoga is a cost-effective resource that can potentially play a role in the pursuit of universal healthcare goals [15]. Consequently, it has become more imperative to assess the potential of integrating yoga into the existing treatment methods for migraines by examining the available evidence.

## Review

Types of headaches and their pathogenesis

Primary headache disorders are classified into different types depending on the cause and pathogenesis of each. In general, the pathogenesis of these headaches ranges from serotonin dysregulation and vasodilation of intra-cranial vessels to increased sensitivity of nociceptive pain fibers. Talking about cluster headaches, a type of trigeminal autonomic cephalgia, no clear evidence regarding the root cause exists; the most accepted mechanism involves activation of the hypothalamus. These signals then trigger the trigeminal-autonomic reflex, leading to the perception of pain and other associated symptoms [16]. Another alternative theory states that it could be due to neurogenic inflammation of the internal carotid artery caused by nerve fibers innervating the intracranial part of the internal carotid artery [17]. Tension headaches occur most likely due to increased sensitivity of central and peripheral nociceptive fibers. These fibers may be activated by electrical, thermal, or pressure stimuli. Furthermore, increased muscle tension has been proposed as a plausible cause for tension headaches, which may irritate nociceptors in the cranial and neck muscles [18]. Table 1 shows the different headache types, their typical duration, and characteristics [19-21].

**Table 1:** Types of migraines and headaches. Sources: Refs. [19-21]

Migraine/Headache Type	Typical Duration	Characteristics
Migraine with aura	4-72 hours	Visual disturbances like flashing lights, followed by headache
Migraine without aura	4-72 hours	Headache pain on one or both sides of the head
Vestibular migraine	Hours to days	Dizziness, vertigo, balance issues
Menstrual migraine	2-3 days	Linked to menstrual cycle, nausea, light sensitivity
Hemiplegic migraine	Hours to days	Weakness on one side of the body, with a headache
Chronic migraine	≥15 days/month	Frequent headaches, nausea, light & sound sensitivity
Ocular migraine	Minutes to hours	Vision loss or disturbances in one eye
Abdominal migraine	Hours to days	Abdominal pain in children, often without head pain
Tension headache	30 min - 7 days	Pressing/tightening (non-pulsating) pain on both sides of the head

Migraine headaches have a diverse and multifactorial pathogenesis. The aura of migraines is believed to be caused by a phenomenon called cortical spreading depression (CSD), in which self-propagating waves of neuronal depolarization are triggered and spread across the cerebrum [22]. These waves lead to the activation of the trigeminal afferent pathway, which, in turn, leads to the release of vasoactive peptides from the trigeminal ganglion; the most significant of them is the calcitonin gene-related peptide (CGRP), which results in neurogenic inflammation and vasodilation, which is thought to be the major cause behind pain experienced in migraines [23]. CGRP increase has also been associated with a decline in descending inhibitory processes, which may lead to migraine vulnerability via sensitization of various central neuronal pathways [24].

CSD is characterized by a slow, spreading wave of depolarization that moves across the cortical tissue. Initially, there is a rise in extracellular potassium (K+) that constantly depolarizes neurons for roughly 30-50 seconds, causing a disturbance of ionic gradients across the cell membrane, an influx of sodium (Na+) and calcium (Ca2+), and the release of glutamate [25]. The hyper-excitable neurons of the cerebral cortex undergo repeated depolarization and repolarization as a result of extracellular potassium accumulation [26]. CSD is the electrophysiological basis for migraine aura and is the cause of the sensory and visual disturbances that often precede a migraine attack. Neurons and glial cells in the affected areas of the brain temporarily depolarize during CSD, causing a disturbance in the ordinary working of neurons and leading to changes in neuronal action [27]. CSD also leads to a change in the permeability of the blood-brain barrier via activation and upregulation of matrix metalloproteinases [28]. A recent literature review has also shown that microRNA may play a role in the pathogenesis of migraines [29]. Pathogenesis of migraines may also be related to the abnormal activation of certain regions of the brain, particularly in the brainstem. These regions comprise locus coeruleus, raphe nuclei, and peri-aqueductal grey matter. The hypothalamus is also thought to be abnormally activated during migraine attacks and could be a possible trigger for this pathology [30]. Lastly, there is also a possible genetic component to the pathogenesis of migraines; a genome-wide association analysis has identified two susceptible loci for migraines without aura [31]. International Classification of Headache Disorders (ICHD-3) has defined and classified all known headache disorders in an algorithmic way [32].

Migraine headache

Migraines are episodic, complex, and familial conditions. Attacks can last up to 72 hours and consist of four overlapping phases [33]. During the first phase, termed the premonitory phase, patients experience non-painful symptoms that appear hours to days before migraine onset [34]. These symptoms can include neck stiffening, fatigue/tiredness, excessive yawning, difficulty concentrating, and mood swings. Some patients also report an increased thirst and an increase in micturition frequency [34]. In the second phase, the aura phase, a subset of patients suffer local neurologic symptoms [35]. Auras are reported mostly by women suffering from migraines, approximately one-third of women experience auras. Auras may occur before or during migraine attacks, and the most common are visual auras, followed by sensory and language [35]. The third phase, headache, is the result of the trigeminal sensory pathway activation, resulting in the throbbing pain characteristic of migraines. The intensity of migraines either progresses throughout the day or is explosive at onset, resulting in the disruption of daily activities [36]. Migraines typically get worse with head movements and can be associated with nausea, vomiting, phonophobia, photophobia, osmophobia, and allodynia. During the fourth phase, postdrome, patients report drowsiness, fatigue, concentration difficulties, and phonophobia or heightened sensitivity to sound. This phase is alternatively termed as a “migraine hangover” among patients [37].

A migraine is diagnosed when patients experience at least five episodes characteristic of migraine headaches. In adults, untreated attacks typically last around four or more hours [38]. For diagnostic purposes, at least two of four of the following features must be present: unilateral pain, pulsatile/throbbing sensation, moderate-to-severe pain usually rated more than five out of 10 by patients, and increased pain intensity with certain movements, such as bending over. Furthermore, one of the following must be associated with the headache: nausea or vomiting, increased sensitivity to light, and increased sensitivity to noise [38,39]. The classification is dependent on the number of days the patient experiences headaches. Episodic migraines are defined as 14 or fewer headaches occurring per month. Chronic headaches on the other hand typically present for more than 15 days a month. The most accurate way of determining the number of days is by determining the number of headache-free days in a specific month [38]. If headaches occur for more than 15 days, then this is termed as chronic headache, and if headaches occur for less than 15 days, then this is termed episodic headache. Patients suffering from episodic headaches are at risk of transitioning to chronic headaches [39]. This usually occurs when both genetic predisposition and environmental factors are present; when combined, they reduce the threshold for migraine attacks and, thus, increase the risk of transitioning to chronic headaches. Environmental factors that may contribute to this include increased stress, increase in body weight, or depression [38,39].

Neuroimaging is not necessary for a diagnosis if the patient's presentation is consistent with migraine headaches [40]. However, it may be ordered when patients report a significant change in the frequency, intensity, or characteristics of the headache [40]. Additionally, if patients present with confusion and report the migraine as the most severe migraine, when the migraine is associated with motor symptoms, or late-life occurring migraine, neuroimaging may be conducted. In addition, if the migraine occurs with a brainstem aura, or the aura occurs without typical migraine characteristics neuroimaging can be conducted in this case as well [35,40].

Yoga

The National Institutes of Health (NIH) classifies yoga, a 3,000-year-old tradition, as an alternative/complementary medicinal approach. It is recognized as a holistic approach to health in Western countries [41]. Yoga, a Sanskrit-originated term, historically represents the fusion of the physical body with the consciousness of the mind and spirit [42]. The basis of yoga philosophy and practice is found in Patanjali's classic work, the Yoga Sutras, which is regarded as the canonical work on the subject. There are different components of yoga, including physical postures, meditation, breathing techniques, lifestyle, and diet. Among them, asanas (posture) are considered one of the most important tools in healing a person. Patanjali also describes ‘eight limbs’ in his Yoga Sutras, also known as ashtanga, which means unification of mind and body. These limbs of yoga practice result in higher states of ethics, spirit, and healing all at once [43,44]. Yoga is a form of mind-body fitness as it consists of a combination of muscular activity with a mindful focus on breathing techniques, self-awareness, and energy. Regular yoga practice cultivates qualities of friendliness, compassion, and self-control while working on improving individual strength, endurance, and flexibility. It also fosters a sense of peace and well-being [44]. Constant exercise also produces significant results, including a more positive life perspective, self-awareness, and an increase in vitality to live life to the fullest with an increased level of happiness and satisfaction [45].

Yoga and meditation are widely recognized as non-pharmacological methods used to improve overall personal health and reduce stress and anxiety levels, chronic pain, headache, and obesity. Various studies have shown that stress is the leading cause of chronic conditions and non-communicable diseases. It is imperative to lessen the impact of disease by focusing on the management of stress. Yoga is a type of complementary and alternative medicine (CAM) that results in a physiological chain of events that reduces the stress response. It is seen as a holistic method for stress treatment. Recent years have seen a substantial rise in scientific research on yoga, and many research studies have been conducted to evaluate its therapeutic effects and advantages. Yoga practices increase physical stamina and flexibility, support and improve respiratory and cardiovascular health, support addiction treatment and recovery, lessen stress, anxiety, and depressive symptoms, promote better sleep, and generally improve well-being and quality of life [46-48]. Table 2 summarizes some of the benefits of yoga versus medications in different aspects of migraine headaches [49-51].

**Table 2:** Comparison between yoga and medication use in migraine headaches. N/A: not applicable Source: Refs. [49-51]

Treatment	Effect on frequency	Effect on pain levels	Effect on disability	Effect on medication use	Other benefits	Limitations
Medications	Abortive medications: reduce the frequency of acute migraines. Preventive medications: reduce the frequency of chronic migraines.	Abortive medications: provide rapid relief. Preventive medications: reduce intensity.	Reduces disability scores.	N/A	Provide rapid acute treatment.	Medications have side effects, include risk of medication overuse.
Yoga	Studies show a reduction in the monthly migraine days.	Studies show a reduction in pain intensity but may take several weeks to improve pain.	Reduces disability scores.	Can reduce the number of pills needed for treatment.	Reduces stress and anxiety, improves muscle tension, low cost, and easy accessibility.	Requires long term practice. Optimal duration remains unknown.

As mentioned previously, migraines have a genetic predisposition as well as a likelihood for environmental factors to play a role. The presence of modifiable and non-modifiable risk factors provides space for alternative therapies such as yoga. Factors such as stress, obesity, diet, sleep patterns, and overuse of medication are among the modifiable risk factors [52]. Conventional medications and treatments aim to relieve symptoms. However, it is vital to address the underlying issue that is causing migraines. In addition, medication use is associated with several side effects, including headaches, memory impairment, and weight gain among others [53]. There are specific side effects, such as gastrointestinal bleeding associated with non-steroidal anti-inflammatory drugs, liver damage associated with acetaminophen, addiction associated with opioid use, depression that has been linked to topiramate, and amitriptyline that has been noted to trigger cardiac arrhythmias and seizures. Given these issues, there has been a growing need for alternatives that are both safe and effective. Addressing stress has a significant role in headaches as it affects the onset, duration, progression, and transition of headaches. Stress is believed to play a role in the sensitization of pain receptors and increase headache-associated risk factors such as sleep patterns, obesity, and diet. Therefore, reducing stress plays a key role in the reduction of headaches, and yoga is beneficial in reducing stress [54]. Table 3 summarizes some of the commonly used medications, their mechanism of action, dose, advantages, and side effects [55-58].

**Table 3:** Commonly used medications for migraine. Sources: Refs. [55-58]

Drug	Mechanism of Action	Dose	Advantage	Side Effects
Sumatriptan	Serotonin 5-HT1 Receptor agonist	50-100 mg oral or 6 mg subcutaneous/intranasal	Rapid relief, effective for acute migraine attacks	Nausea, dizziness, chest tightness
Rizatriptan	Serotonin 5-HT1 Receptor agonist	5-10 mg oral	Rapid relief, effective for acute migraine attacks	Nausea, dizziness, drowsiness
Eletriptan	Serotonin 5-HT1 Receptor agonist	20-40 mg oral	Rapid relief, effective for acute migraine attacks	Nausea, dizziness, drowsiness
Ergotamine	Serotonin 5-HT1 Receptor agonist	1-2 mg oral or sublingual	Effective for acute migraine attacks	Nausea, dizziness, drowsiness, vasoconstriction
Propranolol	Beta blocker	20-160 mg oral daily	Preventive, decreases migraine frequency	Fatigue, dizziness, depression
Topiramate	Inhibits excitatory neurotransmission	25-200 mg oral daily	Preventive, decreases migraine frequency	Paraesthesia, fatigue, cognitive effect

Latest systematic reviews and meta-analysis findings 

A study conducted by Anheyer et al. [59] revealed the presence of a statistically significant effect of yoga in the management of headaches regarding their frequency, duration, and pain intensity. Study findings indicated that yoga may play a role in reducing frequency, duration, and pain intensity in both episodic and chronic headaches, especially immediately after the intervention. Assessing the effect of yoga on different headache types was inconclusive, especially in migraine headaches as the intervention did not yield any statistically significant results in the measured outcomes. It is, however, worth mentioning that the studies did not mention a follow-up duration; therefore, the long-term effect of yoga remains unknown. In addition, none of the included clinical trials reported information on side effects, so no statement can be made regarding safety. The study concluded that yoga is effective in the short-term improvement of headache frequency (p=0.03), duration (p=0.02), and pain intensity (p<0.01) in patients with tension headaches and that further studies are needed to draw deeper connections. The study does have a few limitations, including the small overall population number, high heterogenicity, and poor quality of methodology in the included trials. These limitations do reduce the quality of evidence [59].

Another study conducted by Wu et al. was conducted to measure the efficacy of yoga on migraines. The study included five randomized clinical trials with a total of 356 patients. In comparison to the control group, yoga significantly reduced headache frequency (p=0.0004) and headache impact severity (HIT-6) (p=0.02) [51]. HIT-6 is a questionnaire-type test used to help patients communicate the severity of their headaches and limitations that occur in normal activity due to the negative effects of headaches [60]. Wu et al. did not find any relationship between yoga and pain intensity (p=0.05) or to the McGill Pain Questionnaire (p=0.34) [51]. The McGill Pain Questionnaire consists of three classes of word descriptors used to describe sensory, affective, and evaluative pain experiences [61]. The meta-analysis concluded that adjuvant yoga therapy can add benefits to migraine headaches by reducing headache frequency. The study did have some limitations, including the small sample size and significant heterogeneity in the studies [51]. A study to measure the effectiveness of yoga in migraine headaches, conducted by Long et al. [62] confirmed some of the findings concluded by Wu et al. [51]. The study included six randomized clinical trials. Results found that, when compared to the control group, yoga significantly reduced pain intensity in patients with migraines, and there was a significant heterogeneity in the studies. One of the included papers, Burch et al. [63], was later excluded as its results were out of range and were the probable cause of heterogeneity. After its exclusion, the results indicated that yoga significantly reduced the intensity of pain (p=0.01) and no heterogeneity remained. Regarding secondary outcomes, yoga significantly reduced the frequency of headaches (p=0.0004), duration (p=0.01), HIT-6 score (p=0.003), and migraine disability assessment score (MIDAS) (p<0.0001) [62]. MIDAS is a brief tool used to score lost days because of migraine headaches over three months, including work, school, household work, chores, and social and leisure activities [63].

Previous literature has indicated that yoga improves vagal tone and reduces sympathetic activity, therefore improving autonomic function [64]. Yoga's benefit in migraine patients is related to these two mechanisms as well as improved cardiac autonomic balance [64]. It also increases the levels of nitric oxide, which alleviates migraines by acting as an endothelium-derived relaxing factor-like substance [65]. Yoga does have an impact on migraine headaches, as well as helps reduce pain in the head, neck, and temporal area which are considered trigger zones for migraine headaches. It also helps loosen stiffened muscles, which can act as a trigger for migraine headaches [66]. Yoga also improves the connection between the mind and the body, which makes those who practice it more aware of their body’s sensations and more capable of detecting discomfort or tension giving more time to take preventive measures before the full development of a migraine headache [66]. Evidence-based clinical practice guidelines published in 2023 aimed to provide physicians with a series of recommendations regarding exercise prescriptions for migraine patients [67]. Yoga obtained a grade B recommendation, meaning that it “might” help improve patients’ symptoms. Yoga, including its breathing and relaxation techniques, proved effective in reducing migraine frequency, disability, pain intensity, and attack duration. It is suggested that a minimum six-week program applied three times a week is beneficial for episodic migraines [67]. Further studies are necessary to better understand the duration that a patient would need to participate in yoga to see the benefits.

Latest clinical trial findings

Kumar et al. [68] also confirmed that yoga was an effective add-on therapy and was superior to medical therapy alone. CONTAIN, a prospective open-label randomized superiority clinical trial with blinded endpoints, was conducted in India. The study participants included adults aged 18-50 years previously diagnosed with episodic migraine headaches. Participants were randomized into either the predesigned one-hour yoga program or the medical-only group. The trial used a predesigned yoga intervention for three months and included patients aged between 18 and 50 years of age with a diagnosis of episodic migraine [68]. Both groups had similar baseline and headache characteristics apart from headache frequency noted to be higher in the group receiving yoga add-on therapy. In both groups, propranolol and amitriptyline were the most used prophylactic medication, and acetaminophen and a naproxen-domperidone combination was used as rescue medication [68].

The main endpoints of the study included headache frequency, intensity, and HIT-6 score. The MIDAS score, pill count, and the proportion of participants that were headache-free were among the secondary outcomes of the study. Both groups showed improvement regarding primary and secondary study outcomes. Although patients randomized to the yoga group had a higher headache frequency at baseline, they showed remarkable improvements after the three-month follow-up period. Analysis showed that yoga, in addition to medication, reduced headache frequency (p<0.00001), intensity (p<0.0004), HIT score (p<0.00001), MIDAS score (p<0.00001), and the number of medical pills needed to manage migraines (p<0.00003) when compared to the medication-only group [68]. Although outcomes gradually improved in both groups, the yoga group showed a more consistent speedy improvement compared to the medication-only group. In addition, a higher percentage of participants in the yoga group were headache-free after the follow-up period, this was defined as a lack of need for acute migraine medication, and the calculated number needed to treat (NNT) for this group was eight patients. Regarding side effects, three participants in the medication-only group reported side effects, such as weight gain and dry mouth. In the yoga group, only one participant reported weight gain. More importantly, none of the participants experienced headaches, nausea, or vomiting during the yoga session [68]. 

Another study was conducted by Wells et al. [69] to determine if mindfulness-based stress reduction (MBSR), including yoga improved migraine outcomes and cognition in comparison to headache duration. Most of the included participants were female with a mean age of 43.9 years (with a standard deviation of 13 years). The study aimed to assess the change in headache frequency from baseline to a 12-week point. Secondary outcomes included measuring quality of life self-efficacy, depression scores, alterations in disability, pain intensity, and unpleasantness at 12, 24, and 36 weeks [69]. Participants were followed up for 36 weeks, and the study concluded that both groups had a noticeable reduction in migraine days by week 12 with no significant difference between the two (p=0.50) [69]. However, those participating in MBSR continuously showed improvements in the measure of disability (overall 5.92-point improvement) at all follow-up points. They showed improved quality of life (5.1-point improvement), self-efficacy (8.2-point improvement), catastrophizing pain (5.8-point improvement), and depression score (1.6-point improvement). There was a 36.3% reduction in pain intensity and 30.4% reduction in unpleasantness. In comparison, the headache education group showed an increase in pain intensity (13.5%) and unpleasantness (11.2%) by the end of the trial. This concludes that, although MBSR did not improve migraine frequency, it proved effective in improving other aspects of migraines [69].

## Conclusions

The results of various studies indicate that yoga is effective in helping reduce the clinical symptoms of migraine headaches, along with the associated anxiety and stress, thereby further reducing the aggregation of migraines and their disability. Although there remain several uncertainties regarding yoga, it is a promising non-pharmacological intervention, and further research may provide more insight. In addition, yoga aligns with the WHO's vision of encouraging the development of traditional knowledge that is cost-effective and can serve as a universal medicine. To ensure that best medical practices are established for managing patients, further research is necessary to provide further light on the duration of intervention needed to produce an effective reduction in migraine-associated symptoms.
